# Esthetic Rehabilitation of Anterior Teeth with Copy-Milled Restorations: A Report of Two Cases

**DOI:** 10.1155/2017/2841398

**Published:** 2017-02-23

**Authors:** Sapna Rani, Jyoti Devi, Chandan Jain, Parul Mutneja, Mahesh Verma

**Affiliations:** ^1^Department of Prosthodontics, ITS Dental College, Ghaziabad, India; ^2^Department of Prosthodontics, MAIDS, Delhi, India

## Abstract

Digitalization has become part and parcel of contemporary prosthodontics with the probability of most of the procedures being based on the digital techniques in the near future. This digital revolution started in the latter half of the 20th century by converting analog objects/signals into digital bits and bytes. Recent developments in all-ceramic materials and systems of computer-aided designing and computer-aided manufacturing (CAD/CAM), copy milling, and so forth offer excellent esthetics and superb biocompatibility. Copy milling system for ceramics enables milling of the zirconia cores of all-ceramic restorations precisely and also if this system is properly used the procedure for fabricating all-ceramic restorations can be substantially simplified. This case report presents fabrication of all-ceramic Maryland Bridge and post-core with a copy milling system for esthetics and preservation of integrity of tooth. For both of the patients, the use of biologic, all-ceramic, copy-milled restorations resulted in clinical success and recovered function and esthetics.

## 1. Introduction

Digitization has become an integral part and parcel of contemporary dentistry. New era has shifted to digital dentistry at each step beginning from patient education till final restoration. Initially, an impression was thought to be made of either alginate or rubber base impression material but introduction of computer and laser technology has enabled capture of digital impressions [1]. The digital dentistry provides endless treatment options with enhanced approach, minimum time, and reduced chances of error [2]. Early 1980s paved the way for computer-aided design/computer-aided manufacturing (CAD/CAM) technology. The advantages of this technology are decreasing laboratory work, automation by machine, and production of similar multiple restorations while the disadvantages include technique sensitivity and expensive unit. To overcome disadvantages copy milling was introduced due to its economic set-up and reduced human error [3, 4].

In past time CAD/CAM and copy milling were supposed to be the same but this is not true. CAD/CAM stands for computer-aided designing and computer-aided milling while copy milling is manually designed and computer machined restorations. The information for CAM phase comes from scanning wax/composite coping or framework. Besides the lower cost factor for these types of milling machines, this method of milling allows the dental technician to correct any discrepancies found in the tooth preparation by compensating during waxing of the pattern.

Copy milling is based on pantographic principle that is used to duplicate keys at hardware shops. Using exact mechanical-tactile model surveying and analogous milling [5], it is considered to be highly precise. First, a coping or framework is manually fabricated in wax or composite, and then the pattern is placed into the pantographic machine. The copying arm of the machine traces the wax pattern while the cutting arm, which has a carbide cutter, mills a selected presintered zirconia block. The final shape is 20% to 25% larger to account for shrinkage during the sintering step. The zirconia block has a density barcode label, so the copy mill machine can be adjusted properly to allow for shrinkage during the sintering phase. With the proper use of this system, the procedure for fabricating restorations can be substantially simplified. The simplicity of use of copy milling system, in comparison with complicated software in CAD/CAM technology, makes it a common system for laboratory applications [4]. Zirconia has been widely used as a biomaterial by CAD/CAM technology and also copy milling technique.

Zirconia as a biomaterial was introduced in 1998; yttrium-oxide is added to zirconia in order to stabilize the tetragonal form at room temperature. In today's generation, excellent esthetics and biocompatibility of yttria oxide zirconia based all-ceramic material meet all demands of an ideal prosthetic and restorative material. Yttrium stabilized zirconia has high flexural strength and fracture toughness. Yttrium-oxide partially stabilized zirconia (Y-TZP) has adequate chemical and dimensional stability and its radiopacity makes it easier to distinguish marginal integrity and caries [6]. Due to absence of metal, finishing line at the level of gingiva provides optimal esthetics. In 1990s first copy milling system came into the market, that is, Celay/Cerec, but later on different CAD/CAM systems developed their copy milling units.

Patients not only are becoming more demanding with regard to esthetics but also are often opting for more conservative and less invasive procedures (i.e., more tooth preservation). Successful restoration of dentition depends on three fundamental principles: mechanical preparation to achieve retention and resistance, hence ensuring longevity; aesthetic factors such as minimizing the appearance of margins and display of metal; and the biological consequences of achieving the first two factors which concern the health and ultimate durability of the tooth and periodontium.

The purpose of this clinical report is to present functional and esthetic restoration techniques through the use of copy milling all-ceramic restorations to meet biologic and functional requirements of patients.

## 2. Case Reports

### 2.1. Case  1

**Figure 1:**
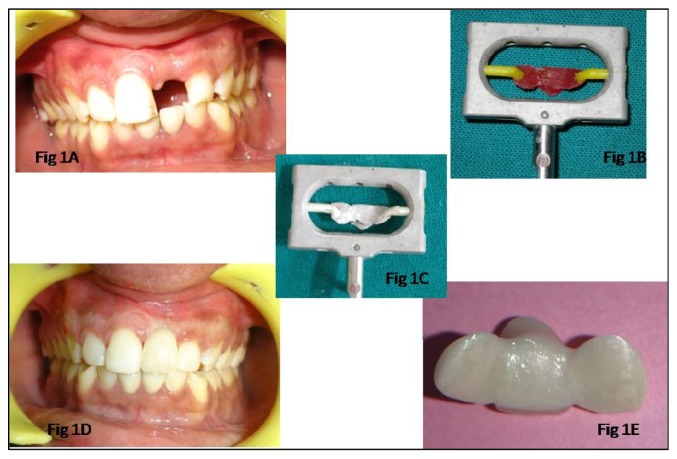
Missing central incisor restored with copy-milled all-ceramic Maryland Bridge. (A) Preoperative photograph showing missing left central incisor. (B) Pattern resin attached to scan holder. (C) Scan spray on pattern resin. (D) Maryland Bridge cemented. (E) Maryland Bridge framework veneered with ceramic.

A young male patient reported to the Department of Prosthodontics with missing left central incisor (Figure 1(A)). On intraoral examination, wear facets were present on the teeth due to the habit of using abrasive tooth powder for brushing. Treatment options available for the patient were porcelain fused to metal (PFM) fixed dental prosthesis (FDP), all-ceramic FDP, and PFM or zirconia Maryland Bridge or implant placement followed by PFM or all-ceramic crown. Although overjet and overbite were not ideal for fabrication of Maryland Bridge, patient insisted on a conservative option which would require lesser amount of tooth preparation. He was unwilling to go for implant therapy because of financial constraints. After taking consent from the patient, rehabilitation of central incisor was planned with all-ceramic copy-milled zirconia Maryland Bridge framework and porcelain layering with a compatible ceramic.

*Procedure.* Tooth preparation was done on right central incisor and left lateral incisor for Maryland Bridge. 1 mm of incisal enamel was left intact and light chamfer finish line was given 1 mm supragingivally. To provide mechanical retention form for the lingual wings, a box preparation was made on the lingual and interproximally leaving buccal line area contact. It was assessed that the occlusal clearance was minimal, so the desired area was then reduced by 0.5 mm with butt-joint cavosurface walls.

Elastomeric single step impression was made using putty and light body silicone (Aquasil, Dentsply) and model was poured in die stone (Ultrarock, Kalabhai). Separating media were applied on the prepared tooth surfaces and pontic area. First wings of the framework were fabricated using pattern resin (GC Corp., Tokyo, Japan) and then pontic area was built up in increments using a small painting brush to give a desired shape and 2 mm of gingival clearance.

After framework has gained initial strength it was carefully retrieved from the model and the pattern was attached with sprue wax (Cercon Wax, Dentsply, Germany) to the respective holder (Figure 1(B)). Later whole assembly was sprayed with scan spray (Cercon, Dentsply, Germany) for copy milling (Figure 1(C)). After milling, zirconia Maryland Bridge framework was separated from blank with straight fissure bur and sintered in Cercon furnace at 1350°C for 6 hours (Cercon Heat, Dentsply, Germany).

Fit of the framework was checked with occlusal spray (Okklean, DFS) and clearance with the opposing arch was assessed. The pontic was then veneered on the labial and incisal surfaces with zirconia Layering Ceramic (Degudent, Dentsply) to give a life-like appearance (Figures 1(D) and 1(E)). After necessary occlusal adjustments, pretreatment of zirconia framework was done with 40 *μ*m alumina, dried with alcohol, and teeth were treated with 37% orthophosphoric acid for 30 sec. Zirconia Maryland Bridge was cemented with adhesive cement (RelyX Unicem; 3M ESPE, St. Paul, Minn) according to manufacturer's instructions. Extra cement was removed with explorer and final restoration was found to have good esthetic and functional value.

### 2.2. Case  2

**Figure 2:**
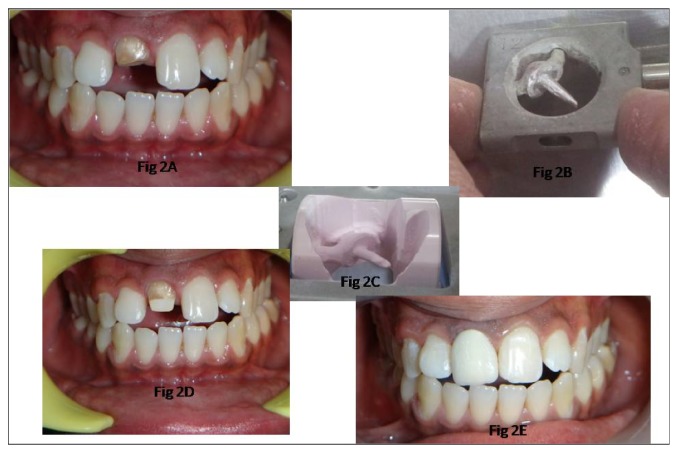
Fractured central incisor restored with copy-milled all-ceramic post and core. (A) Preoperative photograph showing fractured right central incisor. (B) Pattern attached to scan holder. (C) Zirconia framework after milling. (D) Zirconia framework cemented to central incisor. (E) All-ceramic fixed restoration.

A 29-year-old male patient reported to the Department for Rehabilitation of right central incisor. On examination it was found that central incisor was endodontically treated and crown was dislodged (Figure 2(A)). Treatment options available for the patient were metal post and core supported by porcelain fused to metal crown, all-ceramic post with core build-up, and all-ceramic crown and completely milled all-ceramic post-core as one unit supported by all-ceramic crown. After consent from the patient, rehabilitation of central incisor was planned using copy-milled zirconia post and core supported by zirconia based all-ceramic FDP.

*Procedure.* Post space was prepared in the conventional manner [7], assuring that the pulpal axial line angle was rounded and not sharp. Small diameter autopolymerizing acrylic resin (DPI RR Cold Cure, Dental Products, India) dowel was fabricated to make impression of post space. Vaseline was applied in the post space with paper points so that dowel could be relined with pattern resin (GC Corp., Tokyo, Japan) and core build-up was done with the same pattern resin. Pattern was assessed so that there were no sharp angles internally or externally. The pattern was attached with sprue wax (Cercon Wax, Dentsply) to the respective holder (Figure 2(B)) and sprayed with scan spray (Cercon, Dentsply, Germany) for scanning for copy milling. After milling, zirconia post and core was separated from blank with straight fissure bur and sintered in Cercon furnace (Figure 2(C)). Fit of post and core was checked with occlusal spray (Okklean, DFS) and seated completely in the canal space. Post-core was cemented with adhesive cement (RelyX Unicem; 3M ESPE, St. Paul, Minn) according to manufacturer instructions (Figure 2(D)); margins of the preparation were refined for central incisor as necessary for all-ceramic restorations. A two-step putty wash polyvinylsiloxane impression of the prepared tooth was made and poured in type IV gypsum. The die was scanned in Cercon eye and zirconia coping was milled from Y-TZP machinable zirconia block and sintered. The milled unit was then veneered on the labial, incisal, and palatal surfaces with ceramic to give a life-like appearance. Zirconia all-ceramic full-coverage FDP (Cercon, Dentsply) was cemented with adhesive cement (RelyX Unicem; 3M ESPE, St. Paul, Minn) (Figure 2(E)). Extra cement was removed with scalpel and all exposed margins were finished. Final restoration was evaluated clinically and radiographically and appeared to give good esthetic and functional value.

## 3. Discussion

Contemporary dental practice provides endless options for preservation of oral health and for attaining natural esthetics. Digitization has influenced the dental fraternity with the use of computers and scanning methods which has reduced the time for fabrication of restoration. The burnout oven and casting machine have been replaced by model scanning and milling machines.

It is a conflicting topic of discussion that copy milling is at an edge over CAD/CAM. Some literature evaluated that copy milling all-ceramic restorations provided less marginal adaptation as compared to CAD/CAM restorations [8]; however others analyzed that copy milling system may produce more accurate zirconia restorations [9]. Copy-milled restorations in both cases showed good marginal adaptation. In the present case report stabilized zirconia is used for copy milling because of its properties and high survival rate. In dentistry, highest fracture toughness, a high Weibull modulus, and considerable flexural strength (900 Mpa to 1200 Mpa) and great esthetics have made zirconia ceramics a promising material for anterior as well as posterior restorations [10].

Cercon system was used to copy mill restorations in presented case reports and partially sintered zirconia blocks are used in this system. There is less wear of milling instruments in this system as compared to milling of sintered blocks which further minimizes chances of error due to wear of milling burs. Industrially fabricated zirconia blocks lead to increased fracture strength as compared to other ceramics if the shear component of the total load is substantial, as is typically found with anterior teeth. Stabilized zirconia had been chosen for the restoration keeping in mind wear pattern of patients existing dentition which directs the need for stronger material for restoration.

A missing tooth in anterior region is not only a physical loss but also an emotional deprivation for the patient. Restoring a single missing anterior tooth has been a challenge for the dentist as various aspects need to be considered (shade, morphology, gingival contours, and occlusion) [11]. Fixed dental prosthesis may be an aggressive treatment plan when involving tooth preparation of adjacent abutments. Implant therapy is an expensive treatment option and moreover presence of wear facets on dentition is not indicated for implant therapy. The implication of Maryland Bridge prosthesis for the above-mentioned patient with proper treatment plan can serve as a shelter from ill effects related to edentulous space and invasive replacement procedures like fixed dental prosthesis and implants. Metal reinforced Maryland Bridge complicates shade matching because of the shade change that generally occurs on the abutments at cementation due to the cast-metal lingual retainers that impart a gray appearance in the incisal third. The technique described in this article had the advantages of good color match and reduced chances of debonding due to use of adhesive technique. It also allows preservation of tooth structure and makes periodontal assessment easier [12].

The choice of an appropriate restoration for fractured anterior tooth is guided by strength and esthetics. Zirconia copy-milled post and core was chosen for the presented patient as one-piece milled zirconia post and core showed sufficient mean load to failure values for anterior restorations [13]. High elastic modulus makes them less likely to fail adhesively during mastication and permits a more conservative root canal preparation, decreasing the chances of root fracture [14]. Problems encountered in the fabrication of post and core was attachment of sprue to the wax pattern and nonpassive fit of the one-piece milled zirconia post and cores. Nonpassive fit was due to zirconia particles attached to dowel part as scan spray powder particles are also scanned along with the post and core wax pattern and milled from block which requires trimming of post and core to fit in canal space.

## 4. Clinical Significance

Clinician decision should be based on available materials and patient's demands. Copy-milled restorations are a viable treatment option especially when meeting esthetic need of patient.
